# An Experience-Sampling Study on Academic Stressors and Cyberloafing in College Students: The Moderating Role of Trait Self-Control

**DOI:** 10.3389/fpsyg.2021.514252

**Published:** 2021-05-04

**Authors:** Bingping Zhou, Ye Li, Yun Tang, Wentao Cao

**Affiliations:** ^1^Key Laboratory of Adolescent Cyber Psychology and Behavior (CCNU), Ministry of Education, Wuhan, China; ^2^School of Psychology, Central China Normal University, Wuhan, China

**Keywords:** cyberloafing, experience-sampling, academic stressor, self-control, college students

## Abstract

Student cyberloafing is a relatively new educational phenomenon and is getting to be an outstanding issue that educators have to face. It is necessary to find out important factors that aggravate cyberloafing. Using an experience sampling method, this study examined the relationship between academic stressors and cyberloafing. Once a week for five consecutive weeks (T1–T5), 134 undergraduate students assessed the extent of academic stressors and cyberloafing of that week through an electronic questionnaire. Additionally, participants completed a trait self-control scale at Time 2. Results of two-level regression analysis showed that academic stressors were negatively associated with cyberloafing at the within-person level (i.e., week-to-week changes), but not at the between-person level. Furthermore, this relationship pattern was only observed in students with low trait self-control, while those with high trait self-control were less likely to cyberloaf regardless of academic stressors. These findings suggest that cyberloafing can fluctuate over periods, especially for individuals who lack self-control. Future research should consider cyberloafing from a dynamic perspective of individual-context interaction. Several practical implications are also discussed.

## Introduction

Today, no one will doubt the popularity of the Internet and its extensive impact on human life. IPSOS (2016) conducted a poll in a sample of 18,180 people across 23 countries and found that more than two-thirds of people could not imagine life without the Internet. Perhaps this is especially true for college students, since they are more connected to the Internet for various academic activities than ever before, such as course selection, document retrieve, data inquiry and even notes taking (Taneja et al., 2015; Ravizza et al., 2017). Although the Internet has certain benefits, most of the time, labor and leisure are natural enemies, requiring tradeoffs in our cognitive control (Kool and Botvinick, 2014). Students often have to make choice between sticking to learning tasks and pursuing more desirable leisure activities. Internet access to the educational settings is necessary in the present era, but it can easily become a temptation to students. Indeed, students often deviate from task goals during study hours and use the Internet to engage in study-unrelated activities. This phenomenon is known as student cyberloafing (or cyber-slacking) and reflects the “dark side” of Internet usage in the learning environment (Gerow et al., 2010; Taneja et al., 2015).

Cyberloafing is primarily investigated in workplace settings (Lim, 2002; de Lara, 2007; Blanchard and Henle, 2008; Wagner et al., 2012; Moody and Siponen, 2013; Askew et al., 2014; Askew and Buckner, 2017; Lim et al., 2018). With the increased application of information and communication technology (ICT) in the learning environment and the popularity of private digital devices among students, some researchers even pointed out that the extent of student cyberloafing is far higher than that of employees (Akbulut et al., 2017). Compared with the rich research results obtained in workplace settings, cyberloafing research in educational settings is still in its infancy, which is mainly composed of descriptive and cross-sectional studies, and focuses its negative consequences (Ravizza et al., 2017; Wu et al., 2018); see a recent review by Flanigan and Kiewra (2018). Nevertheless, research on the causes and antecedents of student cyberloafing is relatively inadequate. The researchers mainly explored the effects of various demographic factors and some relatively stable psychological variables, which do not play crucial roles in exploring the potential occurrence and development mechanisms of cyberloafing. For example, Baturay and Toker (2015) investigated the impact of several demographics (i.e., gender, grade, Internet skills, Internet usage, and Internet experience) on cyberloafing among 282 high school students; Taneja and colleagues examined the combined effects of multiple psychological factors on student cyberloafing, including intrinsic and extrinsic motivation, class engagement, consumerism, escapism, lack of attention, anxiety, attitude, norms, and perceived behavioral control (Taneja et al., 2015). Thus, it is necessary to further explore factors that influence student cyberloafing from a more dynamic perspective.

In essence, student cyberloafing belongs to a kind of behavior phenomenon in the learning situation, so the most direct antecedent should also be related to learning. Academic stressors are not only closely related to learning, but also one of the most common factors affecting students’ behavior (Kohn and Frazer, 1986; Hurst et al., 2013), which include writing term papers, taking tests, and the constant pressure of studying (Hystad et al., 2009). They are usually related to self-regulation (Oaten and Cheng, 2005). Therefore, it is reasonable that student cyberloafing could also be dependent on academic stressors. This seems to have been overlooked by previous researchers. However, according to previous studies and theories, there seem to be two diametrically opposite patterns of the relationship between the two.

### Academic Stressors and Cyberloafing: Facilitation or Inhibition?

The first is what we call the *stressors-facilitation hypothesis*. According to the general strain theory, a person is more likely to experience negative emotions and subsequently engage in deviant or addictive behaviors upon faced with a high level of strains (Agnew, 1992). In this vein, when facing more academic stressors and having to deal with various learning tasks, students would perceive more emotional distress and then look for ways to get rid of them. Cyberloafing is an effective way to escape from negativity, in part because of its “recovery” impact (Lim and Chen, 2012). To wit, academic stressors may increase cyberloafing. Besides, this hypothesis also echoes the strength model of self-control (Baumeister et al., 1998, 2007; Muraven and Baumeister, 2000). Completing multiple academic tasks and coping with stressors require self-control, and after such efforts, subsequent attempts at resisting the temptation to go online are likely to fail. Imagine that after running out of energy to finish assignments, a student might engage in more cyberloafing in the class. Thus, engaging in non-learning-related Internet activities during study periods can be considered as a corrective strategy to cope with academic stressors. In line with the *stressors-facilitation hypothesis*, prior research has demonstrated that job stressors were positively associated with workplace cyberloafing (RuningSawitri, 2012; Koay et al., 2017). Whether this is the case in educational settings remains to be tested.

Conversely, the other possible form is the *stressors-inhibition hypothesis*. As mentioned above, more academic stressors may lead to more cyberloafing via some mechanism, while perhaps a simpler case is that when coming across heavy academic tasks, students have to put down their mobile phones and put energies into studies in order to meet those academic requirements. Furthermore, stressors usually have a double-edged effect; they both consume psychological resources and enhance motivations and assist individuals in fulfilling responsibilities (Pearsall et al., 2009; Li et al., 2015), thereby reducing redundant actions unrelated to the current task. In plain terms, academic stressors could decrease cyberloafing, on the one hand, due to a lack of leisure time, on the other hand, due to the enhancement of learning motivation.

So far, we have proposed two possibilities for the relationship between academic stressors and cyberloafing, which will be tested in this study. Because academic stressors are variable in a semester, we consider it necessary to adopt an experience-sampling method to address this issue. Moreover, cyberloafing has been studied mainly as a between-person phenomenon, so the previous research methods are primarily cross-sectional surveys. Such a one-time measurement approach offers an inadequate understanding of the within-person fluctuations of cyberloafing in daily experience. Hence, adopting an experience-sampling method could also address the limitations of previous studies that measure cyberloafing as stable behavior, when in fact cyberloafing might be largely situational. In summary, the first aim of the current study was to test whether the *stressors-facilitation hypothesis* or the *stressors-inhibition hypothesis* was more in line with the facts by applying an experience-sampling method.

What needs to be further pointed out is that there are great individual differences in cyberloafing (Jia et al., 2013), and in the stress literature, individual traits are critical in determining how stressors are viewed and how they affect behaviors (Bolger and Zuckerman, 1995; Connor-Smith and Flachsbart, 2007). From a perspective of individual-context interaction, one’s behavior is also affected by individual traits in addition to situational determinants (Lerner, 2004). Thus, the possible interactive influence of situational academic stressors and individual characteristics on student cyberloafing is worth exploring.

### Individual Differences: The Moderating Role of Trait Self-Control

According to an influential framework of personality and daily stress processes (Bolger and Zuckerman, 1995), personality could affect both exposure and reactivity to stressful events. A recent experience sampling study has examined the association between individual differences in trait self-control and daily stress exposure and reactivity in adolescent youth (Galla and Wood, 2015). Likewise, one moderating trait of the relationship between academic stressors and cyberloafing that might be worth noticing is self-control. Self-control is “the ability to override or change one’s inner responses, as well as to interrupt undesired behavioral tendencies (such as impulses) and refrain from acting on them” (Tangney et al., 2004, p. 271). The bulk of research has demonstrated that self-control predicted good adjustment, better performance, and academic success (Tangney et al., 2004; de Ridder et al., 2012). Individuals with high self-control are thought to be dutiful, disciplined, and goal-oriented (Hofmann et al., 2014). Since cyberloafing often has negative impacts on academic performance and goal achievement and can be classified as a form of academic procrastination, it is conceivable that higher dispositional self-control would lead to less cyberloafing in daily academic activities. The similar association has been demonstrated by existing research evidence in the workplace (Restubog et al., 2011; Wagner et al., 2012). Recent evidence showed that individuals with better self-control made better progress on goals by cultivating beneficial habits (Galla and Duckworth, 2015). As such, students with high self-control, who have predispositions to set higher goals and strive for achievement, should be immune to the influence of daily academic stressors, because they are easier to form good and stable study habits, seldom cyberloafing when doing learning tasks. Furthermore, drawing on the strength model of self-control, higher self-control generally means more cognitive resources and less likely to be exhausted by stressors (Muraven and Baumeister, 2000), thus may function as a natural buffer between academic stressors and cyberloafing.

Put differently, the significant relationship between academic stressors and cyberloafing may only be observed among those students with low self-control, regardless of whether this relationship is positive or negative as mentioned before.

### Objectives of the Study

**FIGURE 1:**
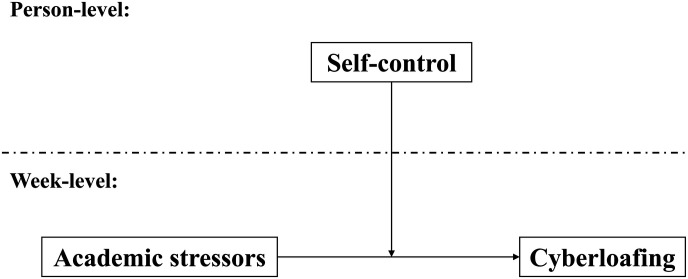
The moderating role of trait self-control in the relationship between academic stressors and cyberloafing.

To further develop the understanding of cyberloafing in educational settings, the current study used an experience-sampling survey to examine a multilevel moderated model, in which academic stressors affected cyberloafing and this effect was different between students with high and low trait self-control (see Figure 1). On an exploratory basis, we assessed the level of academic stressors and cyberloafing for each student on a week-by-week basis (5 weeks in total) so we could test the two opposite hypotheses, namely, whether the within-person fluctuations data supported the stressors-facilitation hypothesis or the stressors-inhibition hypothesis. Besides, we assumed that those students typically with lower trait self-control would be more vulnerable to the effect of academic stressors on cyberloafing.

## Materials and Methods

### Design

As the academic tasks in colleges are generally arranged on a weekly basis, we focused on the process by which academic stressors and cyberloafing wax and wane over weeks. Participants completed a 5-wave experience sampling phase in which we collected descriptions of weekly academic stressors and cyberloafing, as well as self-reported trait self-control. In this way, we could not only know how much variance is within persons and how much is between persons but also observe the covariant relationship between academic stressors and cyberloafing to explain the within-person fluctuations in student cyberloafing.

### Participants

Totally, 134 undergraduate students from local universities who took part in a cross-school psychology degree program were recruited to participate in our study. We initially planned to recruit 150 students based on the standards in the existing literature (Job et al., 2015; Smith and Hofmann, 2016). The final sample size deviates from this target due to our financial and practical constraints. We did not conduct a formal power analysis, because the power analysis of the multilevel linear model is based on simulation and needs to set multiple parameters that quickly increases with model complexity. No analyses were conducted before data collection was completed. Data from all participants who completed at least three surveys and had reported trait self-control were included in the analyses. Thus, the final sample included 534 weeks nested within 110 individuals (91 female) and had a mean age of 19.70 (*SD* = 0.82 years), with the vast majority of sophomores and only three juniors.

Students received partial course credits for their voluntary participation and a bonus (10 CNY) for completing all five questionnaires. The research was approved by the Institutional Review Board of Ethic Committee in Central China Normal University (No. CCNU-IRB-201904-022).

### Procedure

One week before the formal survey, participants provided informed consent and were instructed for the experience sampling phase. All participants began the study in early May and ended in early Jun. Specifically, they were invited to complete an online questionnaire at five-time points (T1–T5), once a week during the second half of the semester. Each week at 22:00 on Saturday night, after they finished their one-week course, we sent the participants a link to the online questionnaire with a request to respond. We would remind them to complete the questionnaire again at 7:30 and close the link at 9:00 on the next day (Sunday) morning. In every questionnaire, participants needed to report cyberloafing and academic stressors during that week. Demographic characteristics were reported at Time 1, and trait self-control was measured at Time 2.

### Measures

#### Cyberloafing

To assess the extent to which college students engage in cyberloafing across weeks, we used a recent scale that was initially developed to measure the general off-task Internet activities at work or study (Guo, 2018). We did not use the existing mainstream scales of cyberloafing in literature because they require participants to report specific Internet activities, such as shopping online, watching videos, checking e-mail (Lim, 2002; Lim and Teo, 2005; Akbulut et al., 2016). This previous approach is not applicable to the current experience-sampling study, since specific Internet activities are vulnerable to some uncontrolled factors caused by time, such as the sudden popularity of some kind of applications at a certain period, or a public event causes most students to follow specific news.

The scale used in the current study required participants to rate the frequency of deliberate Internet use that deviates from tasks and the frequency of unintentional Internet use that unwittingly attracted to the Internet while studying (1 = *Never*, 6 = *Always*). Sample items included “I purposefully complete other tasks that unrelated to study through the Internet” and “I involuntarily went online that unrelated to study” (see Appendix for details). Considering the nested data structure in the current research, we conducted a multilevel confirmatory analysis, using Mplus 7.0 (Muthén and Muthén, (1998-2015)), and the result suggested that this scale also showed a reasonable fit to the data (*χ*^2^ = 167.83, *df* = 68, CFI = 0.95, TLI = 0.93, RMSEA = 0.05). Items were averaged to yield a summary score reflecting cyberloafing, with a higher score indicating more cyberloafing. Cronbach’s α for five weeks ranged between 0.90 and 0.94 (*M* = 0.92).

#### Academic Stressors

The Academic Stress Scale used in previous studies assessed more than 33 academic stressors faced by students (Kohn and Frazer, 1986; Smith and Renk, 2007). Since shortening the scales is an effective way to improve the response rate and lessen the fatigue of participants in experience-sampling research (Uy et al., 2010), we administered five items to assess the most common academic stressors per week for students. The items were: “How much homework did you have this week?” “How many courses did you have this week?” “How many exams did you have this week?” “How many other tasks did you have to complete this week?” “How much leisure time did you have this week (reverse coded)?” Participants responded to these items using a four-point scale (1 = *None*, 4 = *Too much/many*). Items were averaged to yield a summary score reflecting academic stressors, and the higher the score, the more were the academic stressors that participants encountered. Cronbach’s α computed separately for the five weeks ranged between 0.66 and 0.81 (*M* = 0.74).

#### Trait Self-Control

At Time 2 participants completed the revised Chinese version of Self-Control Scale for college students (Tan and Guo, 2008), which was originally developed by Tangney et al. (2004). The revised scale consists of 19 items. Sample items include “I am good at resisting temptation” and “People would describe me as impulsive (reverse coded).” Participants were required to indicate the veracity of each statement when thinking about themselves on a five-point scale (1 = *Strongly disagree*, 5 = *Strongly agree*). Items were averaged to yield a summary score reflecting trait self-control. In the present study, Cronbach’s α for this scale was 0.82.

### Analytic Strategy

Since the weekly data of repeated measurements were clustered within individuals, resulting in a nested data structure, we conducted multilevel analyses by using Mplus 7.0 statistical software (Muthén and Muthén, (1998-2015)). According to the previous recommendations (Enders and Tofighi, 2007), we centered the week-level predictor (weekly academic stressors) at each person’s mean value to appropriately test and interpret the within-person relationship. We also aggregated the score for mean stressors across weeks as a person-level predictor to estimate the effect of academic stressors at the between-person level, albeit initially, we focused on the influence of within-person fluctuations in academic stressors. The person-level variables (trait self-control and mean stressors) were grand-mean centered. The full model is the following:

$${\text{Cyberloafing}}_{\text{ij}}={\text{β}}_{\text{0j}}+{\text{β}}_{\text{1j}}\text{WeeklyStressors}+e_{\text{ij}}$$

$${\text{β}}_{\text{0j}}\text{ = }{\text{γ}}_{\text{00}}\text{ + }{\text{γ}}_{\text{01}}\text{Self-control + }{\text{γ}}_{\text{02}}\text{MeanStressors + }{\text{γ}}_{\text{03}}\text{Self-control × MeanStressors + }{\text{u}}_{\text{0j}}$$

$${\text{β}}_{\text{1j}}\text{=}{\text{γ}}_{\text{10}}\text{+}{\text{γ}}_{\text{11}}\text{Self-control}+u_{\text{1j}}$$

In this model, γ_00_ is the intercept; γ_01_ and γ_0__2_ respectively, represent the main effects of trait self-control and mean academic stressors across weeks on mean cyberloafing; γ_03_ represents the interaction between trait self-control and mean academic stressors at the person-level; γ_10_ represents the main effect of weekly academic stressors on weekly cyberloafing at the week-level; γ_11_ represents the cross-level interaction between trait self-control and weekly academic stressors.

Note that, although we had collected the demographic characteristics of participants (i.e., gender, age and academic level), we did not show them in the main multilevel analyses since they did not substantially alter our results when they were put into our models. This approach is in line with the principle recommended in recent crucial literature on the handling of control variables (e.g., Spector and Brannick, 2010; Becker et al., 2016; Bernerth and Aguinis, 2016) that researchers should be more cautious when considering the inclusion of demographic variables in data analysis, especially when there is not enough theoretical support.

## Results

Table 1 shows the descriptive statistics and correlations for the variables in the study. There was a significant correlation between academic stressors and cyberloafing at the week-level (*r* = –0.11), indicating that cyberloafing changes with the week-to-week fluctuations in academic stressors. As expected, trait self-control was associated with mean cyberloafing at person-level (*r* = –0.26), indicating that the individual differences in trait self-control might lead to the differences in cyberloafing.

**TABLE 1:** Descriptive statistics of and correlations between study variables.

	*M*	*SD*	1	2	3
1. Trait self-control	3.04	0.43	(0.82)	0.09	–0.26**
2. Academic stressors	2.37	0.44	—	(0.74)	–0.05
3. Cyberloafing	3.52	0.86	—	–0.11**	(0.92)

Numbers in parentheses along the diagonal represent the person-level reliability. Numbers above the diagonal are person-level correlations (n = 110). The number below the diagonal is a week-level correlation (n = 534). **p < 0.01.

We conducted three nested multilevel linear models to further investigate the relationships among variables (see Table 2). The null intercept model revealed that 30.1% of the variance in cyberloafing was within-person and 69.9% was between-person. We then entered the main effects of predictors in Model 1. And in Model 2, the product terms of trait self-control with mean stressors at the person-level and with weekly stressors were added to test the interaction effects.

**TABLE 2:** Unstandardized coefficients from multilevel linear models of cyberloafing and affect.

	Null model		Model 1 (Main effects)		Model 2 (Interaction effects)	
Predictors	Estimate (*SE*)	95% CI	Estimate (*SE*)	95% CI	Estimate (*SE*)	95% CI
Intercept	3.51 (0.07)***	[3.37, 3.65]	3.51 (0.07)***	[3.38, 3.65]	3.51 (0.07)***	[3.38, 3.64]
**Week-level**						
Weekly stressors			–0.40 (.09)***	[–0.58, –0.22]	–0.41 (0.09)***	[–0.59, –0.24]
**Person-level**						
Self-control (SC)			–0.45 (0.16)**	[–0.76, –0.14]	–0.47 (0.16)**	[–0.79, –0.16]
Mean stressors			–0.05 (0.19)	[–0.43, 0.33]	–0.03 (0.19)	[–0.41, 0.35]
SC × Mean stressors					0.26 (0.38)	[–0.48, 1.01]
**Cross-level interactions**						
SC × Weekly stressors					0.51 (0.20)*	[0.11, 0.91]
σ^2^	0.22 (0.01)***	[0.19, 0.25]	0.18 (0.01)***	[0.16, 0.21]	0.18 (0.01)***	[0.15, 0.21]
τ_00_	0.51 (0.07)***	[0.36, 0.65]	0.48 (0.07)***	[0.34, 0.61]	0.47 (0.07)***	[0.34, 0.61]
τ_11_			0.28 (0.12)*	[0.04, 0.52]	0.26 (0.11)*	[0.03, 0.48]

Week-level predictor (weekly stressors) is person-mean centered. Mean stressors are measured by averaging individual’s weekly stressors across the study period (five weeks). SE, standard error; CI, confidence interval. *p < 0.05, **p < 0.01, and ***p < 0.001.

In testing main effects (see Table 2), Model 1 shows that weekly academic stressors and trait self-control could significantly negatively predict cyberloafing at the week-level and person-level, respectively, (γ_10_ = –0.40, *SE* = 0.09, *p* < 0.001; γ_01_ = –0.45, *SE* = 0.16, *p* < 0.01); while mean academic stressors across weeks had no such effect (γ_02_ = –0.05, *SE* = 0.19, *ns*). This pattern remains in Model 2 when estimated together with product terms (γ_10_ = –0.41, *SE* = 0.09, *p* < 0.001; γ_01_ = –0.47, *SE* = 0.16, *p* < 0.01; γ_02_ = –0.03, *SE* = 0.19, *ns*). Thus, the *stressors-inhibition hypothesis* was supported.

**FIGURE 2:**
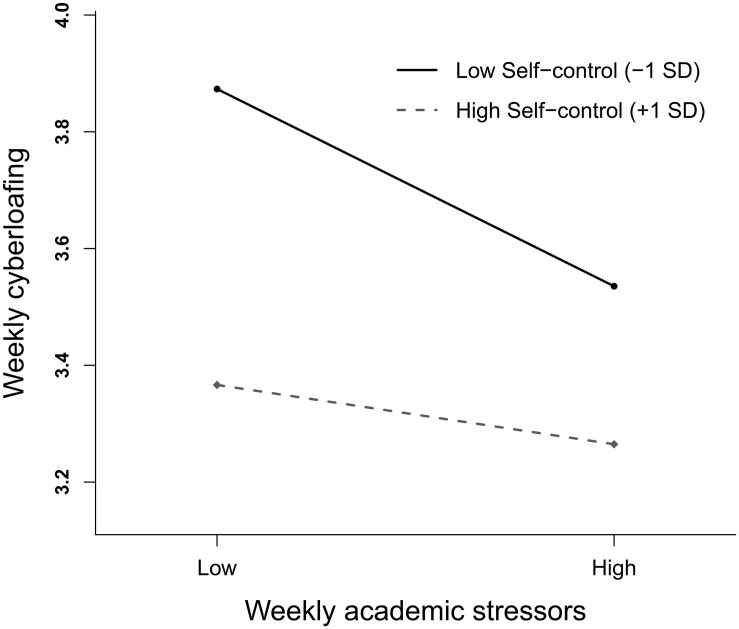
Trait self-control moderating the relationship between academic stressors and cyberloafing (week-level).

In testing the moderation effect, Model 2 shows that the product term of trait self-control with weekly academic stressors was significant in predicting cyberloafing (γ_11_ = 0.51, *SE* = 0.20, *p* < 0.05), while the product term between trait self-control and mean stressors between participants was not (γ_03_ = 0.26, *SE* = 0.38, *ns*). Figure 2 plots the relationship between weekly academic stressors and weekly cyberloafing at low vs. high levels (±1 *SD*) of trait self-control (Preacher et al., 2006). Simple slope tests revealed that weekly academic stressors were negatively related to cyberloafing at a low level of trait self-control (γ = –0.63, *SE* = 0.13, *p* < 0.001), while not related to cyberloafing at a high level of trait self-control (γ = –0.19, *SE* = 0.13, *ns*). This result was consistent with our prior expectation that individuals with low trait self-control would be more vulnerable to the effect of academic stressors on cyberloafing.

## Discussion

To our knowledge, this is the first study using experience-sampling methodology to examine the relationship between academic stressors and cyberloafing in educational settings. Through five consecutive weeks of follow-up surveys, we found that academic stressors were negatively linked to cyberloafing among college students, supporting the *stressors-inhibition hypothesis.* More specially, this negative relationship was observed only at the within-person level (week-to-week changes), but not at the between-person level.

This result is contrary to several previous studies in work filed which have suggested that job stressors were positively related to cyberloafing (RuningSawitri, 2012; Koay et al., 2017). One possible explanation is that the nature of cyberloafing in the work environment is different from that in the educational settings. Cyberloafing in the workplace was generally considered to be a self-regulating way for employees to cope with job stressors (Koay et al., 2017). However, this may not be the case in the educational context. For college students, cyberloafing is more like self-indulgence in the absence of external pressure rather than self-regulation under pressure (Laran, 2010). Compared with work tasks, academic tasks usually have longer periods, are less specific, and place greater emphasis on autonomy, so students are more likely to rely on external or self-imposed deadlines to improve performance (Ariely and Wertenbroch, 2002). When faced with a lot of academic stressors, doing non-learning-related online activities at study time is not a wise strategy to deal with problems. Further research is needed to elucidate this issue.

Another possibility of the discrepancy from previous studies is due to methodological differences. The prior results based on cross-sectional data only reflected the between-person differences, and the stressors and cyberloafing reported by subjects in those studies were relatively stable averages over a period of time. Whereas the primary focus of the current study was on within-person (i.e., week-to-week) changes—whether the degree of cyberloafing varied according to the academic stressors they faced on weeks. Moreover, we found no significant effect after an average of five weeks of academic stressors to predict between-person differences in cyberloafing. This is consistent with a cross-sectional study on non-work presenteeism, which “refers to the behavior of employees who engage in personal activities instead of work-related activities whilst at work” (Wan et al., 2014); it found the levels of job stress were not related to non-work use of ICT. Taken together, we think it is premature to rely on the few current studies to draw conclusions about the relationship between stressors and cyberloafing at the between-person level. First, because the stressors in daily life tends to change, and second, because cyberloafing is easily affected by some stable individual traits, such as the trait self-control (which we will discuss later).

In short, this study considered the fluctuating nature of daily stress and found that weekly academic stressors could negatively predict cyberloafing at the within-person level. Although research evidence points out that daily academic stressors can be detrimental for students both physically and psychologically (Conley and Lehman, 2012; Chung and Cheon, 2017), our research indicated that academic stressors should be very effective in reducing student cyberloafing.

In terms of the between-person variance, it was again demonstrated that individuals with higher trait self-control cyberloaf less while studying, in line with the results of countless studies on self-control and a wide range of behaviors, such as procrastination and cyberloafing (Restubog et al., 2011; de Ridder et al., 2012; Wagner et al., 2012). More intriguingly, we further found that those low in trait self-control were more vulnerable to the influence of daily academic stressors on cyberloafing. Due to its beneficial effects for human functioning and adaption, self-control often plays the role as a protective factor against the impact of situations. For instance, a longitudinal study revealed that more self-controlled children were less likely to become overweight as they enter adolescence, even in today’s obesogenic environment (Tsukayama et al., 2010). Similar results include the moderating effect of self-control on situational variables affecting alcohol use and risky sexual behavior (Quinn and Fromme, 2010), the moderating effect of self-control on sleep quality affecting procrastination (van Eerde and Venus, 2018), and so on. In the current study, students with high trait self-control tend to be immune to the influence of daily academic stressors, corroborating the role of self-control in past work and is in line with our assumptions before. However, the current simple study cannot accurately indicate the mechanism behind this moderating effect, which needs further research data to prove.

Several contributions of our research to the literature are worth noting. First, we enriched the studies of cyberloafing in educational settings, especially tested the impact of academic stressors on this phenomenon, providing a potential reliable interpretation for college student cyberloafing. As we mentioned in the introduction section, several existing studies considered too many influencing factors, making it difficult to delve into a particular mechanism of cyberloafing. Second, the experience-sampling method used in this study provided a reference and enlightenment for future research in cyberloafing domain. In our opinion, cyberloafing should not be seen as just a stable behavior. It can fluctuate over periods, and researchers should pay more attention to its changing nature in daily study and work and observe it from a dynamic perspective. Moreover, many possible influences also have contextual variability, such as moods, stressors and so on. Last but not least, it should be noted that this study explored the two levels of factors (i.e., weekly academic stressors and trait self-control) and their interplay to understand cyberloafing. This approach echoed the current mainstream perspective of individual-context interaction in the field of behavioral research (Lerner et al., 2011).

This study also offers some practical implications. A recent review has proposed a number of instructional strategies for college instructors to curb student cyberloafing inside and outside the classroom (Flanigan and Kiewra, 2018). On the whole, these strategies were carried out from both internal and external aspects, such as enforce technology policies (external) and teach students to self-regulate (internal). Combined with our findings, we speculate that, from the external aspect, one general principle is to properly increase the academic pressure on college students, such as expand the depth of the curriculum and strengthen the assessment of learning process, at least for Chinese universities. From the internal aspect, instructors could help students with low self-control to improve their self-control ability by offering related courses or training programs; students who realize that they usually cyberloaf too much could also take the initiative to develop their self-control ability, for example, engaging in some rituals before attending classroom lectures or doing schoolwork may be helpful (Tian et al., 2018).

## Limitations

We would like to point out some limitations and future directions of the current study. First, all variables were measured via self-report. Although we centered academic stressors at each person’s mean value before data analysis to relieve common method bias, relying on one method of data collection still affects results (Podsakoff et al., 2003). Future work can use more objective indicators such as monitoring apparatus to measure student cyberloafing (Calderwood et al., 2014). Second, the consecutive surveys were conducted on a weekly basis, future research can be conducted on a daily basis and for a longer period of time to confirm the validity of the relationship between stressors and cyberloafing. Third, this study is essentially a correlational study and cannot support a cause-effect conclusion, and due to the lack of mediating variables, it is unable to reveal the mechanism that underlies the relations. Future research should add possible mediating variables, and ensure time intervals of measurements in order to explore the subtle influence process. Fourth, the gender and academic level composition of our sample limits the interpretation and generalization of current results. Future research could use wider and more heterogeneous populations to test the robustness of these findings.

## Conclusion

Student cyberloafing is a relatively new educational phenomenon and is also a thorny problem faced by the educational system. It is urgent to find out the most important factors that could cause cyberloafing. Data from this experience-sampling study suggested that student cyberloafing varied over weeks in a semester and was negatively related to academic stressors. This relationship was only observed in students with low trait self-control, compared with those with high self-control.

## Data Availability Statement

The datasets generated for this study are available on request to the corresponding author.

## Ethics Statement

The studies involving human participants were reviewed and approved by Central China Normal University, Ethic Committee, EC, Institutional Review Board. The patients/participants provided their written informed consent to participate in this study.

## Author Contributions

BZ and YL contributed to the conception and design of the study. YL and WC collected the data. BZ performed the statistical analysis and wrote the first draft of the manuscript. YL and YT critically revised the manuscript. All authors read and approved the submitted version.

## Conflict of Interest

The authors declare that the research was conducted in the absence of any commercial or financial relationships that could be construed as a potential conflict of interest.

## Appendix

Cyberloafing Scale (originally in Chinese, translated by the author).

In the past week, how much did you experience the following when you were studying (1-“never”, 6-“always”):

1. I was vulnerable to the interference of Internet stimuli and put down the task at hand.

2. I involuntarily went online that unrelated to study.

3. My attention was attracted by Internet stimuli.

4. I forgot my tasks inadvertently due to the attraction of Internet stimuli.

5. I unconsciously viewed online information that has nothing to do with study.

6. I used internet to take care of things other than study.

7. I purposefully completed other tasks that unrelated to study through the Internet.

8. I used study time to go online to handle some other private matters.

9. I got online upon convenience and deal with personal matters.

10. I went online to deal with other things as planned.
